# Patellar Tendon Reconstruction With Achilles Allograft: A Technical Note

**DOI:** 10.1177/26350254251368929

**Published:** 2025-12-08

**Authors:** Ahab G. Alnemri, Amar S. Vadhera, Rahul Kumar, Hasani Swindell, Safa Gursoy, Nikhil N. Verma, Jorge Chahla

**Affiliations:** † Perelman School of Medicine, Philadelphia, Pennsylvania, USA; ‡ Sidney Kimmel Medical College, Philadelphia, Pennsylvania, USA; § University of Massachusetts T.H. Chan School of Medicine, Worcester, Massachusetts, USA; ‖ Hoag Orthopedic Institute, Irvine, California, USA; ¶ Division of Sports Medicine, Department of Orthopaedic Surgery, Midwest Orthopaedics at RUSH, RUSH University Medical Center, Chicago, Illinois, USA; Investigation performed at Midwest Orthopaedics at RUSH, RUSH University Medical Center, Chicago, Illinois, USA

**Keywords:** Achilles tendon allograft, allograft surgery, patellar tendon, patellar tendon reconstruction, patellar tendon rupture

## Abstract

**Background:**

Patellar tendon ruptures are relatively common injuries, often caused by high tensile forces on a weakened tendon. In younger patients, they typically result from eccentric loading on the extensor mechanism, whereas in older patients, trauma is the more common cause. These injuries can be functionally limiting and challenging to treat when chronic due to tendon atrophy, scarring, and proximal retraction.

**Indications:**

Reconstruction is indicated for patients with chronic, symptomatic patellar tendon ruptures confirmed by clinical and radiographic evaluation.

**Technique Description:**

A longitudinal midline incision was made, and dissection proceeded through the subcutaneous tissue to identify the ruptured tendon. Fibrotic tissue was debrided, and the tendon length was adjusted to restore patellar height. Three 3.5-mm drill holes were created at the inferior pole of the patella for 5.5-mm bio-composite anchors. Sutures were placed in a Krackow interlocking fashion, with limbs tied in full extension. An Achilles tendon allograft with a bone block was prepared. A 10 × 20 mm tunnel was drilled distal to the tibial tubercle, and the graft was secured with a metal interference screw. The graft was looped proximally and secured to the native tendon with suture tape in a double-layer Krakow configuration. Additional anchors were placed at the tibial tubercle to secure the distal graft.

**Results:**

This technique demonstrated excellent functional outcomes, with radiographic evidence of a healed, well-fixed graft and restored patellar height. The patient achieved 120° of knee flexion, full extension, and independent ambulation at the 6-month follow-up.

**Discussion/Conclusion:**

Achilles allograft augmentation offers a reliable solution for chronic patellar tendon ruptures, especially in cases with tendon atrophy or retraction, eliminating donor site morbidity and enabling strong fixation and early rehabilitation.

**Patient Consent Disclosure Statement:**

The author(s) attests that consent has been obtained from any patient(s) appearing in this publication. If the individual may be identifiable, the author(s) has included a statement of release or other written form of approval from the patient(s) with this submission for publication.

## Graphical abstract

**Graphical Abstract:**
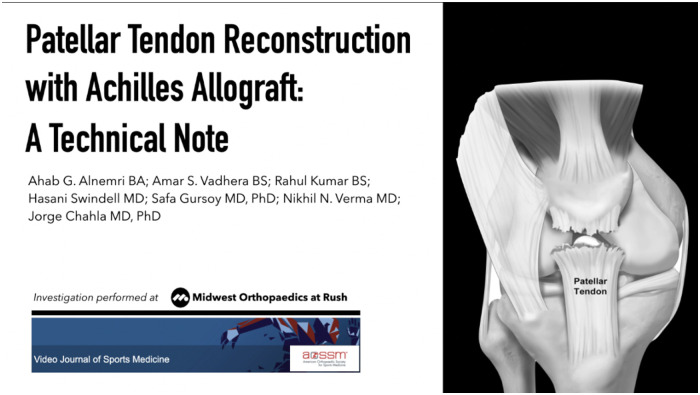
This is a visual representation of the abstract.

## Video Transcript

Here, we present a case of patellar tendon reconstruction with an Achilles allograft.

## Background

The patellar tendon, originating from the inferior pole of the patella and inserting at the tibial tubercle, is responsible for knee extension and is a continuum of the extensor mechanism complex of the knee. Tears to this tendon have been reported to have an incidence of just <1 in 200 people in the United States population per year.^7^ Patellar tendon ruptures can occur as a complication of a total knee arthroplasty^11^ or anterior cruciate ligament reconstruction (ACLR), specifically during a bone–patellar tendon–bone autograft harvest.^1^ When present, reconstruction of the tendon may be necessary if treated after 2 weeks from the time of the initial tear, often augmented with an allograft, and, in rare cases, an autograft.^5^

## Indications

Our patient was a 46-year-old woman with previous ACLR. She had chronic, dull right knee pain after a fall sustained 2 years prior. This pain worsened over time, rated a 6 out of 10, and worsened with knee extension and walking. After failing conservative management, she presented for evaluation of further treatment options.

On examination of the right knee, there was a mild effusion, and passive range of motion was from 2° of hyperextension to 110° of flexion. The active range of motion examination revealed an extensor lag of 30° to 40°. The remainder of the examination was otherwise unremarkable.

Plain radiographs showed hardware from her previous ACLR. On the lateral view, patella alta was noted with ossific densities at the inferior pole of the patella and an Insall–Salvati ratio of >1.2. Given the patient's history, physical examination, and imaging, the diagnosis of patellar tendon rupture was made, and a patellar tendon reconstruction with allograft augmentation was planned to restore her extensor mechanism function and address the insufficient native tendon integrity.

## Technique Description

The patient was intubated, and the operative extremity was sterilely prepared and draped. A longitudinal midline incision was made centered over the patella and extended proximally and distally. Dissection was carried past the subcutaneous tissue, and the chronically ruptured tendon was identified. The fibrotic tissues were debrided, and the mobility of the patella-quad tendon complex was assessed. Adequate excursion of the patella-quad tendon complex was confirmed, so quad tendon mobilization was not required to approximate the patella to the patellar tendon stump.

In similar chronic cases, we have considered a hamstring autograft and V–Y quadriceps advancement. However, these alternatives risk donor site morbidity or inadequate restoration of length. An Achilles allograft with a bone block was selected for its secure tibial fixation, avoidance of autograft harvest, and reliable restoration of patellar height and extensor function.

Three anchors—placed medially, at midline, and on the lateral aspect of the inferior pole of the patella—were used for the reconstruction. This was done while palpating the articular surface of the patella to prevent injury to the cartilage. A 3.5-mm drill was used to create 3 drill holes, followed by placement of 5.5-mm biocomposite anchors using a tap.

The free suture ends from these anchors were passed distally and then proximally through the patellar tendon tissue in a Krakow fashion. The sutures were then passed in alignment with their corresponding anchors along the medial, lateral, and central aspects of the tendon. The matching pairs of medial, central, and lateral suture limbs were then tied together, with the knee in full extension, to reduce and secure the native patellar tendon.

Given the chronicity of the injury, the 30° to 40° of extensor lag, and the degenerative quality of the remaining tendon, primary repair alone was deemed insufficient. An autograft was not selected because of her previous ACLR and the associated donor site morbidity. Therefore, an Achilles tendon allograft was prepared to augment the reconstruction and restore structural integrity to the extensor mechanism. A bone block was fashioned to be placed distal to the tibial tubercle, measuring 10 mm in diameter and 2 cm in length. A guide pin was placed perpendicular to the tibia, just distal to the tibial tubercle, and subsequently overreamed to a depth of 2 cm using a 10-mm diameter reamer. The prepared bone plug was placed into the hole. A flexible nitinol wire was placed at the inferior aspect of the bone plug, and the plug was fixed with a 7 × 20 mm metal interference screw.

Next, the Achilles allograft was laid proximally over the repair and folded so that the proximal extent of the graft overlaid the patellar tendon. Traction stitches were placed at the folded portion of the allograft to maintain this length relationship. Intraoperative tensioning was performed with the knee in full extension to ensure the graft was taut without overconstraint to preserve physiologic tracking. Using suture tape, the allograft was augmented and secured to the native tendon in a Krakow fashion along with the medial and lateral aspects of the graft. The limbs were passed distally and proximally along the graft-tendon construct and subsequently tied. The tied suture tails were cut, and the distal free end of the allograft was excised at the level of the tibial tubercle. An additional 5.5-mm bio-composite anchor was placed at the level of the tibial tubercle, and the suture limbs from the anchor were used to secure the distal extent of the allograft of the tibia. This was performed with sutures passed in a Krackow fashion proximally and distally, followed by tying of the free suture limbs distally.

Final stability was confirmed in 0° to 30° of flexion, and the wound was closed in a standard, layered fashion.

The procedure was completed in approximately 90 minutes—including preparation and fixation of the allograft. Postoperatively, the patient was placed in a brace in extension and allowed to weightbear as tolerated. The brace remained locked in extension until the first postoperative visit at week 2. Physical therapy was initiated on postoperative day 1, with the goal of the patient achieving full extension and 90° of flexion within 6 to 8 weeks. By 6 months, the patient had achieved full extension and 120° of flexion, and had returned to daily activities without the use of assistive devices. At our institution, several similar cases have demonstrated consistent graft incorporation and restoration of extensor function, with minimal complications.

## Results

Results from this procedure have demonstrated excellent restoration of the extensor mechanism with improved quadriceps strength and functional range of motion, enabling many patients to return to full ambulation and daily activities.^2,8,9^ Radiographic follow-up confirmed graft incorporation and normalization of patellar height, supporting durable structural restoration.^8,9^

## Discussion/Conclusion

Biomechanical studies show that Achilles allograft repairs with bone blocks nearly double the load to failure—from just <400 N with only a primary repair to 750 N with an allograft and a bone block—and minimize gap formation, therefore improving construct integrity under stress.^2^ Other studies additionally report sustained clinical improvements in range of motion and extensor function, with minimal to no extensor lag and no major complications at mid-term follow-up.^3,6^ The Achilles tendon allograft remains particularly valuable in chronic patellar tendon rupture cases where the native tissue is incompetent, as the bone block design enables robust tibial fixation and early mobilization. While complications related to allograft use are a concern, a 2019 meta-analysis of 30 studies on extensor mechanism disruption after total knee arthroplasty reported a 3.9% infection rate with allografts, which is comparable to that of synthetic materials. It also observed similar rates of success and revision rates. Furthermore, case series have shown no infections or reruptures with full graft incorporation at 2-year follow-up.^4,11^ For future procedures, using an Achilles tendon allograft with a bone block may enable more precise restoration of native patellar tendon length and patellar height, minimization of extensor lag, and improvement of anatomic knee mechanics.^10^
